# Quality of Life in Patients with Coronary Artery Disease—Multicenter POLASPIRE II Study

**DOI:** 10.3390/jcm13133630

**Published:** 2024-06-21

**Authors:** Józefa Dąbek, Marek Styczkiewicz, Karol Kamiński, Aldona Kubica, Dariusz A. Kosior, Renata Wolfshaut-Wolak, Marek Rajzer, Magdalena Szynal, Piotr Jankowski, Zbigniew Gąsior

**Affiliations:** 1 Department of Cardiology, Faculty of Health Sciences in Katowice, Medical University of Silesia in Katowice, ul. Ziołowa 45-47, 40-635 Katowice, Poland; magdalena.szynal@sum.edu.pl (M.S.); zgasior@sum.edu.pl (Z.G.); 2 Department of Cardiology, Independent Public Provincial Hospital, Jana Pawła II 10, 43-170 Zamość, Poland; styczkiewicz@interia.pl; 3 Department of Cardiology, Medical University of Bialystok, M. Skłodowskiej-Curie 24A, 15-089 Białystok, Poland; fizklin@gmail.com; 4 Department of Health Promotion, Collegium Medicum in Bygdoszcz, Nicolaus Copernicus University in Toruń, Skłodowskiej-Curie 9 Bygdoszcz, 87-100 Toruń, Poland; aldona.kubica@gmail.com; 5 Department of Cardiology, Central Clinical Hospital of The Ministry of Internal Affairs and Administration, Wołoska 137, 02-507 Warszawa, Poland; dkosior13@gmail.com; 6 Institute of Nursing and Midwifery, Jagiellonian University Medical College, Michałowskiego 12, 31-008 Kraków, Poland; rwolfshaut@gmail.com; 7 Institute of Cardiology, Collegium Medicum, Jagiellonian University Krakow, 31-008 Kraków, Poland; rajzer37@interia.pl; 8 Department of Internal Medicine and Gerontology, Medical Center for Postgraduate Education, 01-813 Warszawa, Poland; piotrjankowski.eu@gmail.com

**Keywords:** quality of life, coronary artery disease, POLASPIRE

## Abstract

**Background**: The quality of life of patients with coronary heart disease is extremely important for their treatment. The aim of the study was to assess the quality of life of patients with coronary artery disease, considering education and compliance with medical recommendations regarding lifestyle changes, as well as the presence of selected cardiovascular risk factors. **Methods:** The study involved 763 patients from 11 Polish cardiology centers. The presented material is part of the multicenter POLASPIRE II study. Patients completed a standardized questionnaire EuroQol 5D-5Lm. A medical interview was conducted with each patient. All patients had their body weight and height measured and BMI determined. **Results:** The quality of life of patients was better in men, younger people, those with lower body weight and those who followed preventive recommendations and intensified their physical activity. Most of the examined patients complied with the medical recommendations regarding lifestyle changes after a cardiac incident, but they mainly concerned dietary modifications. There was still a large group of patients who did not comply with the recommendations, e.g., regarding increasing physical activity. **Conclusions:** The assessment of quality of life depended on many factors, such as gender, body weight and compliance with medical recommendations. The health education of patients in the presented study group was not sufficient. Therefore, there is a need for better education regarding the benefits of following medical recommendations in terms of leading a healthy lifestyle, which consequently improves its quality and duration.

## 1. Introduction

According to the World Health Organization (WHO), quality of life is “*an individual’s way of perceiving his or her life position in the cultural context and value system in which he or she lives and in relation to the tasks, expectations, and standards determined by environmental conditions*” [1,2]. Studying the quality of life in the medical concept means identifying problems resulting from the disease and the treatment used and relating to human activity in the physical, mental and social sense and describing the patient’s views on health and their subjective well-being [3,4].

Every year, 17 million deaths occur worldwide due to cardiovascular diseases, and as many as 5 million deaths occur in European Union (EU) countries. The Organization for Economic Co-operation and Development (OECD) published data according to which diseases of the circulatory system were responsible for a much higher share of deaths in Poland compared to the EU in 2019—45%, an increase from 36% in 2016 [5]. The consequences of coronary artery disease include not only a shortening of life but also a significant deterioration of its quality.

Compliance with medical recommendations regarding lifestyle changes plays an important role in the quality of life in cardiac patients. Following a balanced diet to maintain proper body weight, as well as conducting regular physical activity, allows, among others, to prevent the progression of the disease, positively influencing the quality and length of life.

Obesity and overweight are significant risk factors for the onset of cardiovascular diseases, such as coronary artery disease. In 2022, 2.5 billion adults aged 18 years and older were overweight, and 890 million adults were obese [6]. In Poland, however, as many as 58% of adult Poles were overweight, and the result was above the European average of 53% (*Eurostat research* from 2019). By 2050, being overweight may shorten the average life expectancy in Poland by almost 4 years [7]. Another disturbing fact is that as many as 80% of Poles consider overweight only as a cosmetic defect (the *“Let’s talk honestly about obesity” campaign* of 2021) [8]. There are studies in the literature in which obese people declared a worse quality of life than people with normal body weight [9].

Cardiovascular risk factors include little or no physical activity. Regular physical activity has a beneficial effect on increasing the efficiency of the circulatory system, reducing blood pressure, increasing the stroke volume of the heart and improving the elasticity of blood vessels, as well as reducing the risk of developing atherosclerosis and its complications [10,11,12]. According to the literature, regular physical activity also promotes better quality and even extension of life [10,13]. The Ministry of Sport and Tourism’s study on the level of physical activity of Poles in 2018 revealed that only 21.8% of Poles met the WHO’s requirements for physical activity [14].

The aim of the study was to assess the quality of life of patients with coronary artery disease, considering education and compliance with medical recommendations regarding lifestyle changes, as well as the presence of selected cardiovascular risk factors.

## 2. Materials and Methods

The study involved 763 patients from 11 Polish cardiology centers and included patients aged 18–80 years who had been hospitalized in the last 6–18 months due to myocardial infarction (with or without ST segment elevation), unstable angina, percutaneous coronary interventions (PCI) or eligibility for treatment under coronary heart disease surgery (CABG). The study required all patients to consent and be able to complete the survey independently. The study excluded patients who did not consent to participate in the study, were hospitalized for a duration exceeding 18 months or less than 6 months for the above-mentioned reasons and were unable to independently complete the questionnaire. On the day of the examination, the patient’s clinical condition was stable. They did not report any symptoms, including symptoms of angina pectoris or heart failure, as well as significant arrhythmias. There were no deviations from the norm in the blood pressure and heart rate measurements, and no new changes were observed in the resting ECG compared to the tests performed during hospitalization.

The study was conducted from December 2022 to December 2023. The study was started after obtaining the consent of the regional Bioethics Committees (no PCN/CBN/0022/KB1/97/21), and all patients gave informed consent to participate in the study. This material is part of the multicenter POLASPIRE II study.

Patients were recruited in the study based on medical records, considering the inclusion and exclusion criteria listed above. They were invited for a check-up, during which detailed medical history was collected; blood and urine were tested in laboratory tests; an ECG was performed; blood pressure, body weight, body height and the amount of carbon monoxide in exhaled air were measured. Additionally, all examined patients had their body mass index (BMI) determined. They were also asked to fill out a questionnaire containing, among other things, a standardized quality of life assessment questionnaire EuroQol 5D-5L. Before completing the questionnaire on their own, each study participant was informed on precisely how to complete it and that their answers should refer to the situation on the current day of the study. The researchers conducting the study were present when the respondent completed the questionnaire and provided assistance if necessary to ask questions or clarify doubts. Completing the questionnaire was completely voluntary.

The above-mentioned questionnaire considers 5 dimensions of quality of life: mobility, self-care, ability to perform usual activities, feeling pain/discomfort, feeling anxious/depressed. The respondents rated them using a 5-point Likert scale with the following answers: No problems, Slight problems/slight severity, Moderate problems/moderate severity, Serious problems/severe severity and Inability to perform activities/very severe [15]. The discussed questionnaire also includes a visual analog scale (EQ-VAS, EuroQol visual analog scale), scored from 0 (worst possible well-being) to 100 (best possible well-being), with which patients described their health condition. This made it possible to obtain information about how good/how bad a day the subject was having at a given moment [16].

Moreover, a thorough medical interview was conducted with each patient, during which the above-mentioned questionnaire was supplemented with questions about physical activity and compliance with medical recommendations regarding lifestyle modifications before and after the coronary incident.

Statistical analyses were performed using Statistica software ver. 13.1 (Statsoft Poland). The results of the study group were prepared by presenting qualitative data as the number of respondents in individual groups and percentages in relation to the entire population and quantitative data, which considered descriptive statistics, i.e., mean, median, standard and quartile deviation, minimum and maximum value. The distribution of the quantitative data examined was examined using the Shapiro–Wilk test. Due to the lack of normal distribution of the analyzed data, the Mann–Whitney U-test was used to examine the differences between quantitative data in two groups, and the Kruskal–Wallis test was used for analysis of the differences in multiple groups. Due to the lack of normal distribution, Spearman’s rank test was used for correlation analysis between quantitative data. In all the analyses performed, the level of statistical significance was set at *p* < 0.05.

## 3. Results

Table 1 shows the general characteristics of the study group. Most respondents were aged 66–75 (375; 49.15%). Only 133 individuals (17.43%) had normal body weight, while as many as 312 (77.85%) were obese. Slightly more than half of respondents engaged in light physical activity (402; 52.69%).

Table 2 presents the characteristics of the study group, including respondents’ answers regarding obtaining information from medical staff about the need to take the mentioned preventive actions. The surveyed patients were most often informed by medical staff about the need to take preventive actions in terms of changing their diet (larger amounts of fruit and vegetables—406; 51.20%, fat reduction—393; 49.56%, sugar reduction—362; 45, 65%).

Table 3 presents the characteristics of the study group, including respondents’ answers regarding health-promoting behaviors undertaken after a coronary incident. After a coronary incident, significantly more of the examined patients took preventive actions, especially in terms of changing their diet (larger amounts of fruit and vegetables—483; 60.91%, fat reduction—474; 59.77%, sugar reduction—416; 52.46%, salt reduction—415; 52.33%).

Table 4 shows the characteristics of the study group, including the number of preventive actions taken after a coronary incident. Most respondents implemented 6–10 health-promoting activities (305; 39.97%).

Table 5 presents the characteristics of the study group, including descriptive statistics and analysis of differences in the EQ-index, and Table 6 includes information on EQ-VAS depending on gender, age, body mass index, physical activity and the number of health-promoting activities undertaken.

Data analysis showed statistically significant differences in the EQ-index and EQ-VAS values. The EQ-index and EQ-VAS scores were higher in men than in women (*p* = 0.09 and *p* = 0.003, respectively).

The EQ-index decreased with age (*p* < 0.001) and body weight (*p* = 0.001), increased with increasing intensity and frequency of physical activity (*p* < 0.001), and in terms of the number of health-promoting activities undertaken, it was the highest in people who implemented 11–13 of them (*p* = 0.002).

The EQ-VAS was the highest in people aged 46–55 years (*p* < 0.001) declaring intensive physical activity for 20 min 1–2 times a week (*p* < 0.001), and it increased with the number of health-promoting activities undertaken (*p* = 0.009).

Considering body weight, the differences in EQ-VAS points were not statistically significant (*p* = 0.16).

Post hoc analyses (Tables S1–S7—additional material) showed the following differences between individual variables:

(a) between patients with class II obesity and people with reduced and normal body mass index values, as well as overweight and obesity in the EQ-index range; a higher body mass index was associated with a lower quality of life (Table S1),
(b) between people aged 76–80 and people aged 33–45, 46–55, 56–65,
(c) as well as between the ages of 66–75 and 46–55 and 56–65 and 46–55 in terms of the EQ-index; older age was associated with a lower quality of life (Table S2),
(d) between patients implementing 11–13 preventive actions and people implementing 1–5 and 6–10 of them in terms of the EQ-index (Table S3); a smaller number of preventive activities implemented was associated with a lower quality of life,
(e) between respondents who do not engage in physical activity and those who declare light activity, intensive activity for at least 20 min 1–2 times a week and intensive activity for at least 20 min 3 or more times a week, as well as light and intensive activity for at least 20 min 1–2 times a week in the EQ-index range (Table S4); more intensive and frequent use of physical activity was associated with a higher quality of life,
(f) between people aged 46–55 and patients aged 56–65, 66–76 and 76–90, as well as those aged 76–90 and 66–75 in terms of the EQ-VAS (Table S5); older age was associated with a lower assessment of well-being,
(g) between patients implementing 11–13 preventive measures and people implementing 1–5 of them in terms of the EQ-VAS (Table S6); a smaller number of preventive activities implemented was associated with a lower assessment of well-being,
(h) between respondents who do not engage in physical activity and declare that they engage in intensive activity for a minimum of 20 min 1–2 times a week and intensive activity for a minimum of 20 min 3 or more times a week, as well as light activity and intensive activity for a minimum of 20 min 3 or more times a week in terms of the EQ-VAS (Table S7); more intensive and frequent use of physical activity was associated with a higher assessment of well-being.

Table 7 presents the characteristics of the study group, including the analysis of the relationship between quality of life (EQ-index) and self-assessment of health (EQ-VAS) and the values of body mass index, age and the number of health-promoting activities undertaken.

Correlation analysis showed statistically significant negative relationships between the values of body mass index and EQ-index (*p* = 0.003) and age, as well as between the EQ-index (*p* < 0.001) and EQ-VAS (*p* < 0.001). However, positive correlations were found between the number of health-promoting activities undertaken and the EQ-index (*p* < 0.001) and EQ-VAS (*p* = 0.001).

As the body mass index increased, the quality of life deteriorated in the examined patients. A similar relationship was shown considering age, but self-assessment of health also decreased with age.

Both quality of life and self-assessment of health among patients in the study group increased with the number of health-promoting activities undertaken.

## 4. Discussion

In our study, we examined whether patients were adequately informed by medical staff regarding the necessity to adopt preventive measures and whether they actually implemented the above-mentioned actions. In both cases, the surveyed patients most often declared lifestyle modifications in terms of changing their diet, and most respondents implemented from 6 to 10 health-promoting activities.

Analyzing the results in Table 2 and Table 3, it can be seen that the percentage of information obtained from medical staff about the need to take preventive measures (Table 2) and compliance with them by the respondents are similar (Table 3). The study was conducted among patients 6–18 months after a coronary event, i.e., relatively shortly after hospitalization. This may explain why a similar number of respondents said that they had received the above-mentioned recommendations from doctors and remembered which ones they had followed.

It should be noted that only about half of the respondents declared that medical staff informed them about the need to take preventive measures, most often in the field of nutrition (larger amounts of fruit and vegetables—406; 51.20%, fat reduction—393; 49.56%, sugar reduction—362; 45.65%). Considering other variables, the percentage of respondents was even smaller, e.g., intensification of general daily physical exercises—282; 35.56% or reducing alcohol consumption—248; 31.27%. Our research showed that the education of patients with coronary artery disease provided by medical staff was still insufficient. Patient education is significant in the process of treating cardiac patients. I. Kazimierska, in her article on cardiology education, quoted Prof. E. Straburzyńska-Migaj, who said that “a patient who is more aware of his disease and better educated is a patient who is treated more effectively” [17]. Education should include providing basic knowledge about the risk factors and the benefits of eliminating them by changing to a healthy lifestyle. It should also be individualized to the needs of each patient. Medical staff should be obliged to provide information on this subject during each hospital stay or clinic visit, as well as to motivate patients to make changes [18]. A. Waśniowska et al., in their research on the knowledge of risk factors for cardiovascular diseases and the risk of death among middle-aged residents of Krakow, showed a clear relationship between ignorance of the risk factors and a higher risk of death from cardiological causes [19]. A. Ślifirczyk described a study on preventive and educational activities as part of the district health and prevention program “Together for the Heart” among the inhabitants of Biała Podlaska, Poland. The researcher showed that the most common recommendation by doctors working in primary health care clinics (POZ) was a check-up at a cardiology clinic, and this recommendation concerned only 27% of all respondents [20].

An analysis of the differences between the quality of life assessed by the standardized EuroQoL 5D-5L questionnaire and the number of preventive actions undertaken was performed. The EQ-index points were highest in people declaring that they had taken 11–13 actions, and the EQ-VAS points increased with the number of health-promoting actions taken.

This means that the quality of life assessed by the respondents was the highest in people declaring that they took the highest number of health-promoting activities, and health satisfaction increased with the greater number of health-promoting activities undertaken. A correlation analysis of the above-mentioned variables was also performed, and they were statistically significant, both in the case of the EQ-index and the EQ-VAS, meaning that the quality of life and self-assessment of health increased with the number of health-promoting activities undertaken. This showed how important comprehensive, and not just selective, action is in prevention. Moreover, the study revealed the scale of the problem of a large group of patients still remaining who did not comply with preventive recommendations and whose health education was insufficient.

L. Duminova et al. conducted research on a group of 878 patients and showed that men complied with medical recommendations more effectively than women. Their analysis also observed that patients aged 58 and over adhered to medical recommendations more effectively than those below the mentioned age [21]. The study by L. Al- Daken and N. Eshah on patients with hypertension showed that the respondents most often complied with medical recommendations regarding taking medications but were reluctant to follow dietary recommendations (e.g., reduction in sodium intake) or regular check-ups [22]. M. Saki et al. examined the effect of patient-centered education among patients with coronary artery disease on treatment adherence. In their study, they showed a significant difference between the average results of adherence to the treatment regimen in three dimensions: diet, physical activity and medications [23].

Our study also included an analysis of differences in quality of life assessed with the standardized EuroQol 5D-5L questionnaire and gender, age, body mass index (BMI) and physical activity. Correlations of the above-mentioned variables were also included.

Statistically significant differences were observed in the EQ-index and EQ-VAS scores, which were higher in men than in women. On this basis, it can be concluded that the surveyed men assessed their quality of life and well-being as better than women. E. Szudy and G. Chojnacka-Kowalewska assessed the quality of life of 100 patients after a heart attack using the standardized SF-36 questionnaire. However, their study did not demonstrate any relationship between gender and quality of life. Moreover, the mentioned researchers did not find any relationship between age and quality of life in their study group [24].

In our study, the EQ-index decreased with age, and the EQ-VAS was the highest in people aged 46–55. The use of correlation analysis also showed negative statistical significance, indicating that “older” people assessed their quality of life and well-being as worse.

A. Chatzinikolaou et al. assessed the quality of life of patients with ischemic heart disease using three standardized questionnaires, including the EuroQoL5D-5L presented in our study but also the SF-36 and MacNew. Their analysis also found no statistically significant differences between women and men in any quality of life measure, except for the pain subscale of the SF-36, in which men reported a higher quality of life assessment in terms of pain than women [25]. In turn, considering age, in the study cited above, similar results were obtained as in our study, i.e., study participants over 73 years of age obtained a significantly lower EQ-index result than those under 55 years of age (*p* = 0.01), those aged 55–65 years (*p* = 0.01) and those aged 66–72 years (*p* = 0.02), as well as a significantly lower EQ-VAS health assessment result compared to patients under 55 years of age (*p* = 0.03). The results of the remaining questionnaires in the study cited above were similar [25].

According to M. Payne et al., obesity was closely associated with mental health and quality of life [26]. As mentioned in the Introduction, there are reports in which obese people declared a worse quality of life than people with a normal body weight [9]. M. Murray et al., researching a group of adolescents, noticed that overweight or obese people declared a similar quality of life to people with cancer [27]. M. Rybka and I. Kawczyńska assessed the quality of life of people aged 65 and over with obesity using the standardized WHOQOL-BREF questionnaire. They showed that the BMI value was related to the assessment of quality of life and health satisfaction, i.e., overweight and obese people assessed these aspects as worse than people with normal body weight [28]. Our study also confirmed the above-mentioned relationships. The assessment of quality of life differed between people with different body weight; it was lower in patients with increased body weight compared to those with normal body weight. The correlations used also showed a negative relationship between body mass index and quality of life, indicating that overweight and obese people declared a worse quality of life than people with a normal body weight.

According to the literature, regular physical activity has a beneficial effect on increasing the efficiency of the circulatory system, reducing blood pressure, increasing the stroke volume of the heart and improving the elasticity of blood vessels. It also reduces the risk of developing atherosclerosis and its complications [10,11,12].

K. Kontoangelos et al., in their research on risk factors and quality of life (in which, among other things, they assessed the physical capacity of the subjects during a treadmill test), showed that the intensity and duration of exercise were associated with better results among the examined patients obtained in individual subscales regarding the physical components and physical functioning of the SF-36 quality of life questionnaire [29]. V. Katsi et al., in their study on exercise tolerance and quality of life in patients with known or suspected coronary artery disease, showed that good exercise tolerance was associated with better results among the examined patients in the physical and mental health domains in the SF-36 quality of life questionnaire and general HRQL results [30]. M. Staniute et al. conducted an analysis among 1072 patients with coronary artery disease undergoing rehabilitation, in which it was noted that reduced quality of life was associated with greater fatigue and reduced exercise capacity [31]. Our study showed a difference between increasing the intensity and frequency of physical activity and the EQ-index and EQ-VAS: respondents who declared greater intensity and frequency of regular physical activity rated their quality of life and well-being better.

In conclusion, the presented study demonstrated a significant relationship for public health between the use of preventive medicine and the quality of life. The more patients implemented preventive measures, the better the quality of life declared. As mentioned earlier, this demonstrated how important comprehensive, not just selective, action is in prevention. Furthermore, the study group was large, with 763 people from 11 different cardiology centers throughout Poland. This allowed us to reveal the scale of the problem among a large group of patients still remaining who do not follow preventive principles. The results of the study draw attention to the important problem of the need for systematic health education in society.

The study was not free from limitations. Data regarding medical recommendations regarding prevention and its implementation by patients were based on the respondents’ answers, not on medical records. Moreover, the study group included patients both 6 and 18 months after a coronary event with varying degrees of disease progression; therefore, individuals may have shown a greater tendency to follow medical recommendations and declare a better quality of life than others, as the quality of life questionnaire was completed by all respondents on the day of this study, not during hospitalization.

## 5. Conclusions

The quality of life of patients included in the POLASPIRE study was better in men, younger people, those with lower body weight and those who followed preventive recommendations and intensified their physical activity. Most of the examined patients complied with the medical recommendations regarding lifestyle changes after a cardiac incident, but they mainly concerned dietary modifications. There was still a large group of patients who did not comply with the recommendations, e.g., regarding increasing physical activity. The health education of patients in the presented study group was not sufficient. Therefore, there is a need for better education regarding the benefits of following medical recommendations in terms of leading a healthy lifestyle, which consequently improves its quality and duration.

## Figures and Tables

**Table 1:** General characteristics of the study group.

Entire Study Group (763; 100%)			
Variables		n	%
**Sex**	Women	190	24.90
	Men	573	75.10
**Age [years]**	33–45	14	1.83
	46–55	68	8.91
	56–65	205	26.87
	66–75	375	49.15
	76–80	101	13.24
**Body mass index**	Reduced (<18.50 kg m^−2^)	5	0.66
	Normal (18.50–24.99 kg m^−2^)	133	17.43
	Overweight (25.00–29.99 kg m^−2^)	302	39.58
	Obesity I° (30.00–34.99 kg m^−2^)	222	29.10
	Obesity II° (35.00–39.99 kg m^−2^)	70	9.17
	Obesity III° (≥40.00 kg m^−2^)	20	2.62
	No data	11	1.44
**Physical activity**	No physical activity during the week	128	16.78
	Light physical activity in the last week	402	52.69
	Intensive activity for a minimum of 20 min 1–2 times a week	79	10.35
	Intensive activity for a minimum of 20 min 3 or more times a week	154	20.18

**Explanation of abbreviations**: n—number.

**Table 2:** Characteristics of the study group, including respondents’ answers about obtaining information from medical staff about the need to take the mentioned preventive actions.

Entire Study Group (763; 100%)		
Preventive Action	n	%
Eating more fruit and vegetables	406	51.20
Reducing fat intake	393	49.56
Reducing sugar consumption	362	45.65
Reducing caloric intake	355	44.77
Eating more fish	350	44.14
Reducing salt intake	348	43.88
Changing the types of fats you eat	345	43.51
Following dietary recommendations	317	39.97
Increasing overall daily physical exercise	282	35.56
Eating more oily fish	270	34.05
Using exercises recommended by medical staff	266	33.54
Participating in physical exercise	252	31.78
Reducing alcohol consumption	248	31.27
Regular consumption of products enriched with vegetable fats (sterols/stanols)	198	24.97
Attending sports activities or a club	95	11.98
Joining group hikes	96	12.11
Use of weight loss medications	37	4.67

**Explanation of abbreviations**: n—number.

**Table 3:** Characteristics of the study group, including respondents’ answers on the topic of health-promoting behaviors undertaken after a coronary incident.

Entire Study Group (763; 100%)		
Preventive Action	n	%
Eating more fruit and vegetables	483	60.91
Reducing fat intake	474	59.77
Reducing sugar consumption	416	52.46
Reducing salt intake	415	52.33
Reducing caloric intake	403	50.82
Changing the types of fats you eat	397	50.06
Eating more fish	384	48.42
Following dietary recommendations	352	44.39
Increasing overall daily physical exercise	334	42.12
Reducing alcohol consumption	287	36.19
Eating more oily fish	276	34.80
Using exercises recommended by medical staff	248	31.27
Participating in physical exercise	242	30.52
Regularly eating food products enriched with vegetable fats (sterols/stanols)	215	27.11
Attending sports activities or a club	73	9.21
Joining group hikes	68	8.58
Use of weight loss medications	26	3.28

**Explanation of abbreviations**: n—number.

**Table 4:** Characteristics of the study group, including the number of preventive actions taken after a coronary incident.

Entire Study Group (763; 100%)		
Variables	n	%
0	98	12.84
1–5	165	21.63
6–10	305	39.97
11–13	116	15.20
No data	79	10.35

**Explanation of abbreviations**: n—number.

**Table 5:** Characteristics of the study group, including descriptive statistics and analysis of differences in the EQ-index depending on gender, age, body mass index, physical activity and the number of health-promoting activities undertaken.

Entire Study Group (763; 100%)			
Variables		Z/H	*p*
**Sex**	Women	−2.61	0.009 *
	Men		
**Age**	33–45	30.24	<0.001 *
	46–55		
	56–65		
	66–75		
	76–80		
**Body mass index [kg·m^−2^]**	Lowered	20.07	0.001 *
	Correct		
	Overweight		
	Obesity I°		
	Obesity II°		
	Obesity III°		
**Number of health-promoting activities undertaken**	0	14.55	0.002 *
	1–5		
	6–10		
	11–13		
**Physical activity**	No physical activity during the week	29.67	<0.001 *
	Light physical activity in the last week		
	Intensive activity for a minimum of 20 min 1–2 times a week		
	Intensive activity for a minimum of 20 min 3 or more times a week		

**Explanation of abbreviations**: n—number, Z—value of the Mann–Whitney U statistics for two compared groups, H—value of the Kruskal–Wallis statistics for many compared groups, *p*—statistical significance, *—statistically significant result.

**Table 6:** Characteristics of the study group, including descriptive statistics and analysis of differences in the EQ-VAS index depending on gender, age, body mass index, physical activity and the number of health-promoting activities undertaken.

Entire Study Group (763; 100%)			
Variables		Z/H	*p*
**Sex**	Women	−3.01	0.003 *
	Men		
**Age**	33–45	28.92	<0.001 *
	46–55		
	56–65		
	66–75		
	76–80		
**Body mass index [kg m^−2^]**	Lowered	7.89	0.16
	Correct		
	Overweight		
	Obesity I°		
	Obesity II°		
	Obesity III°		
**Number of health-promoting activities undertaken**	0	11.56	0.009 *
	1–5		
	6–10		
	11–13		
**Physical activity**	No physical activity during the week	25.59	<0.001 *
	Light physical activity in the last week		
	Intensive activity for a minimum of 20 min 1–2 times a week		
	Intensive activity for a minimum of 20 min 3 or more times a week		

**Explanation of abbreviations**: n—number, Z—value of the Mann–Whitney U statistics for two compared groups, H—value of the Kruskal–Wallis statistics for many compared groups, *p*—statistical significance, *—statistically significant result.

**Table 7:** Characteristics of the study group, including correlation analyses of the EQ-index and EQ-VAS values, body mass index, age and the number of health-promoting activities undertaken.

Correlations	R	*p*
Body mass index value and EQ-index	−0.110	0.003 *
Body mass index value and EQ-VAS	−0.050	0.167
Age and EQ-index	−0.160	<0.001 *
Age and EQ-VAS	−0.134	<0.001 *
Number of health-promoting activities undertaken and EQ-index	0.148	<0.001 *
Number of health-promoting activities undertaken and EQ-VAS	0.124	0.001 *

**Explanation of abbreviations:** R—Spearman’s rank correlation value, *p*—statistical significance value, *—statistically significant result.

## Data Availability

Data available on request only for scientific purposes.

## Supplementary Materials

The following supporting information can be downloaded at https://www.mdpi.com/article/10.3390/jcm13133630/s1, Table S1: Post hoc analysis of differences considering the values of patients’ body mass index and EQ-index; Table S2: Post hoc analysis of differences considering the age of patients and EQ-index; Table S3: Post hoc analysis of differences considering the number of health-promoting actions undertaken and EQ-index; Table S4: Post hoc analysis of differences considering physical activity and EQ-index; Table S5: Post hoc analysis of differences by age category and EQ-VAS; Table S6: Post hoc analysis of differences considering the number of health-promoting actions undertaken and EQ-VAS; Table S7: Post hoc difference analysis considering physical activity and EQ-VAS.
