# Supplementary material for: Quality of Life in Patients with Coronary Artery Disease—Multicenter POLASPIRE II Study
Source: J Clin Med. 2024 Jun 21;13(13):3630. doi: 10.3390/jcm13133630 (PMC11242409; doi:10.3390/jcm13133630)
Supplement: Supplementary file 1 [file jcm-13-03630-s001.zip › jcm-3050253-supplementary.pdf]

Supplementary material to the article entitled:

Quality of Life in Patients with Coronary Artery Disease—Multicenter POLASPIRE II Study

Supplementary Tables S1–7 (S1-S7) contain post-hoc statistical analyzes constituting additional material to the article with the title mentioned above.

Table S1. Post-hoc analysis of differences considering the values of patients' body mass index and EQ-index

Body mass index value	Reduced	Correct	Overweight	Obesity I°	Obesity II°	Obesity III°
**Reduced**	-	NS	NS	NS	**0.029**	NS
**Correct**	NS	-	NS	NS	**0.003**	NS
**Overweight**	NS	NS	-	NS	**0.013**	NS
**Obesity I°**	NS	NS	NS	-	**0.037**	NS
**Obesity II°**	**0.029**	**0.003**	**0.013**	**0.037**	-	NS
**Obesity III°**	NS	NS	NS	NS	NS	-

Explanation of abbreviations: NS – result not significant

Table S2. Post-hoc analysis of differences considering the age of patients and EQ-index

Age - years	33-45	46-55	56-65	66-75	76-90
**33-45**	-	NS	NS	NS	**0.021**
**46-55**	NS	-	**0.034**	**0.001**	**<0.001**
**56-65**	NS	**0.034**	-	NS	**0.045**
**66-75**	NS	**0.001**	NS	-	NS
**76-80**	**0.021**	**<0.001**	**0.045**	NS	-

Explanation of abbreviations: NS – result not significant

Table S3. Post-hoc analysis of differences considering the number of health-promoting actions undertaken and EQ-index

Number of health-promoting activities undertaken	0	1-5	6-10	11-13
**0**	-	NS	NS	**0.003**
**1-5**	NS	-	NS	**0.014**
**6-10**	NS	NS	-	**0.032**
**11-13**	**0.003**	**0.014**	**0.032**	-

Explanation of abbreviations: NS – result not significant

Table S4. Post-hoc analysis of differences considering physical activity and EQ-index

Physical activity	No physical activity during the week	Light physical activity last week	Intensive activity for min. 20 minutes 1-2 times a week	Intensive activity for min. 20 minutes 3 or more times a week
**No physical activity during the week**	-	**0.011**	**<0.001**	**<0.001**
**Light physical activity last week**	**0.011**	-	**0.026**	NS
**Intensive activity for min. 20 minutes 1-2 times a week**	**<0.001**	**0.026**	-	NS
**Intensive activity for min. 20 minutes 3 or more times a week**	**<0.001**	NS	NS	-

Explanation of abbreviations: NS – result not significant

Table S5. Post-hoc analysis of differences by age category and EQ-VAS

Age - years	33-45	46-55	56-65	66-75	76-90
**33-45**	-	NS	NS	NS	NS
**46-55**	NS	-	**<0.001**	**0.001**	**<0.001**
**56-65**	NS	**0.001**	-	NS	NS
**66-75**	NS	**0.001**	NS	-	**0.032**
**76-80**	NS	**<0.001**	NS	**0.032**	-

Explanation of abbreviations: NS – result not significant

Table S6. Post-hoc analysis of differences taking into account the number of health-promoting actions undertaken and EQ-VAS

Number of health-promoting activities undertaken	0	1-5	6-10	11-13
**0**	-	NS	NS	NS
**1-5**	NS	-	NS	**0.044**
**6-10**	NS	NS	-	NS
**11-13**	NS	**0.044**	NS	-

Explanation of abbreviations: NS – result not significant

Table S7. Post-hoc difference analysis considering physical activity and EQ-VAS

**Physical activity**	**No physical activity during the week**	**Light physical activity last week**	**Intensive activity for min. 20 minutes 1-2 times a week**	**Intensive activity for min. 20 minutes 3 or more times a week**
**No physical activity during the week**	-	NS	**0.003**	**<0.001**
**Light physical activity last week**	NS	-	NS	**0.017**
**Intensive activity for min. 20 minutes 1-2 times a week**	**0.003**	NS	-	NS
**Intensive activity for min. 20 minutes 3 or more times a week**	**<0.001**	**0.017**	NS	-

Explanation of abbreviations: NS – result not significant
